# Monitoring Microwave Ablation Using Ultrasound Echo Decorrelation Imaging: An ex vivo Study

**DOI:** 10.3390/s19040977

**Published:** 2019-02-25

**Authors:** Zhuhuang Zhou, Yue Wang, Shuang Song, Weiwei Wu, Shuicai Wu, Po-Hsiang Tsui

**Affiliations:** 1 College of Life Science and Bioengineering, Beijing University of Technology, Beijing 100124, China; zhouzh@bjut.edu.cn (Z.Z.); wangyue_0603@buaa.edu.cn (Y.W.); ssjiayou.happy@163.com (S.S.); 2 College of Biomedical Engineering, Capital Medical University, Beijing 100054, China; wuweiwei8889@163.com; 3 Department of Medical Imaging and Radiological Sciences, College of Medicine, Chang Gung University, Taoyuan 33302, Taiwan; 4 Medical Imaging Research Center, Institute for Radiological Research, Chang Gung University and Chang Gung Memorial Hospital at Linkou, Taoyuan 33302, Taiwan; 5 Department of Medical Imaging and Intervention, Chang Gung Memorial Hospital at Linkou, Taoyuan 33302, Taiwan

**Keywords:** microwave ablation, ablation zone, instantaneous echo decorrelation imaging, cumulative echo decorrelation imaging, thermal lesion, ultrasound radiofrequency signal, monitoring

## Abstract

In this study, a microwave-induced ablation zone (thermal lesion) monitoring method based on ultrasound echo decorrelation imaging was proposed. A total of 15 cases of ex vivo porcine liver microwave ablation (MWA) experiments were carried out. Ultrasound radiofrequency (RF) signals at different times during MWA were acquired using a commercial clinical ultrasound scanner with a 7.5-MHz linear-array transducer. Instantaneous and cumulative echo decorrelation images of two adjacent frames of RF data were calculated. Polynomial approximation images were obtained on the basis of the thresholded cumulative echo decorrelation images. Experimental results showed that the instantaneous echo decorrelation images outperformed conventional B-mode images in monitoring microwave-induced thermal lesions. Using gross pathology measurements as the reference standard, the estimation of thermal lesions using the polynomial approximation images yielded an average accuracy of 88.60%. We concluded that instantaneous ultrasound echo decorrelation imaging is capable of monitoring the change of thermal lesions during MWA, and cumulative ultrasound echo decorrelation imaging and polynomial approximation imaging are feasible for quantitatively depicting thermal lesions.

## 1. Introduction

Hepatocellular carcinoma (HCC) is one of the most common malignant tumors [1]. Currently, surgical resection is considered to be the first choice for the treatment of HCC. However, due to factors such as the location and size of the tumor and the presence of blood vessels around the tumor, only a few patients are suitable for surgical resection. In recent years, thermal ablations have become an effective treatment for liver tumors. The physical mechanism is that the tissue temperature is raised to above 60 °C by heating, which leads to coagulation necrosis of cancer cells, thus killing them. Thermal ablations have the advantages of minimal invasiveness and short recovery time after the procedure. At present, the main techniques of thermal ablations are radiofrequency ablation (RFA), microwave ablation (MWA), high intensity focused ultrasound (HIFU), and laser ablation [2]. Intra-operative monitoring of ablation zones (coagulation zones) plays an important role in maximizing the killing of tumor cells and protecting the normal liver tissues during ablation.

The major imaging techniques of tumor ablation monitoring are magnetic resonance imaging (MRI) [3], ultrasound imaging [4], and computed tomography (CT) [5]. MRI has a high accuracy in measuring tissue temperature during ablation, but its real-time performance is not high, and the cost is expensive. CT causes ionizing radiation to human body, which is not suitable for long-term monitoring. Recently, microwave tomography has also been proposed as a tool to monitor thermal ablation treatments [6]. Ultrasound imaging has been widely used because of its noninvasiveness, real-time capability, and low cost. However, conventional B-mode ultrasound imaging is susceptible to the influence of gas bubbles generated during ablation, which produces artifacts below the bubbles (posterior acoustic shadow), preventing the ablation zone from being accurately depicted [7]. Ultrasound echo decorrelation imaging traces ablation-induced tissue degeneration by calculating the decorrelation of two adjacent frames of ultrasound backscattered radiofrequency (RF) signals. Subramanian [8] pointed out that compared with other quantitative ultrasound techniques, the ultrasound echo decorrelation imaging technique is easier to use to achieve real-time monitoring of tumor ablation. 

At present, the ultrasound echo decorrelation imaging technique is mostly used in ablation monitoring of HIFU and RFA. Using non-HIFU ablation experiments (*n* = 12) and HIFU ablation experiments (*n* = 21) of bovine liver tissue ex vivo, Fosnight et al. [9] demonstrated that ultrasound echo decorrelation imaging can predict cell death in real time. Fosnight et al. [10] showed that ultrasound echo decorrelation imaging is an effective method for monitoring HIFU ablation of rabbit liver VX2 tumors in vivo (*n* = 13). Abbass et al. [11] investigated the feasibility of real-time control of HIFU ablation using echo decorrelation imaging feedback. According to the preliminary HIFU ablation experiments of ex vivo bovine liver tissue (*n* = 13), the minimum cumulative decorrelation image threshold (log_10_-scaled echo decorrelation per millisecond) was selected as −2.7, and the specificity of coagulation zone detection could reach 90%. Matsuzawa et al. [12] and Sasaki et al. [13] demonstrated that significant decorrelation was observed in the focal spot of HIFU exposure. The feasibility of the ultrasound echo decorrelation imaging technique in RFA monitoring has also been proved. Mast et al. [14] conducted RFA (*n* = 9) on fresh bovine liver tissue ex vivo, collected RF signals, and performed echo decorrelation imaging. The results showed that the echo decorrelation imaging could show the changes of tissue after RFA. The local echo decorrelation was closely related to the increase of local tissue temperature and the effect of thermal ablation. Subramanian et al. [15] investigated the correlation between echo decorrelation coefficient and temperature by simulating the temperature field in RFA (*n* = 3) of liver ex vivo, and verified the potential capability of echo decorrelation imaging for thermal ablation monitoring. The above work shows that ultrasound echo decorrelation imaging can track and quantify the changes of ultrasound RF signals to predict RFA-induced thermal lesions ex vivo. Subramanian et al. [16] showed the feasibility of ultrasound echo decorrelation imaging as a RFA monitoring tool using in vivo experiments (*n* = 7). Hooi et al. [17] theoretically studied the effects of an ultrasonic measurement system, RF signal window, electronic noise, and tissue motion on the decorrelation image, and proposed a method to reduce motion artifacts. The theoretical analysis was verified by simulation experiments and in vivo experiments of porcine liver RFA. These indicate that the feasibility of ultrasound echo decorrelation imaging in RFA monitoring in vivo has been further demonstrated. However, the feasibility of ultrasound echo decorrelation imaging in monitoring MWA remains unknown.

In this paper, the feasibility of ultrasound echo decorrelation imaging in monitoring MWA was explored. An ex vivo porcine liver ablation model was designed. Ultrasound RF signals were collected during the MWA procedure. These signals were analyzed to construct B-mode ultrasound and instantaneous echo decorrelation images for monitoring MWA. Finally, the cumulative echo decorrelation image and the polynomial approximation were calculated to quantitatively evaluate the ablation zone. Experimental results showed that instantaneous echo decorrelation imaging is capable of monitoring porcine liver MWA ex vivo, while cumulative echo decorrelation imaging and polynomial approximation can quantitatively describe the thermal lesions.

## 2. Materials and Methods

2.1. Experimental Setup

**Figure 1:**
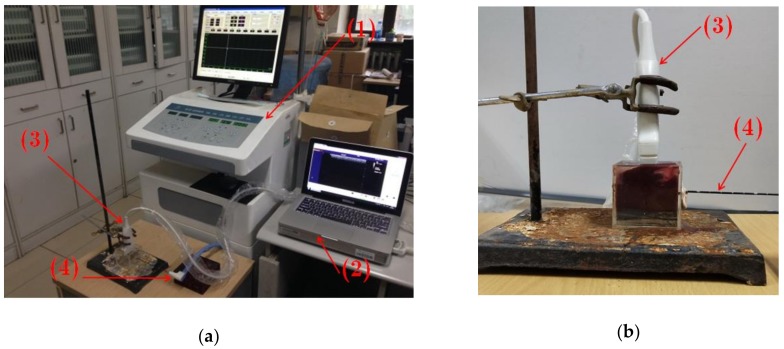
Experimental setup. (**a**) General experimental setup. (**b**) Close-up of the acrylic case with a sample of porcine liver. (1): Microwave ablation device; (2): Ultrasound scanner; (3) Ultrasound transducer; (4): Microwave ablation needle.

Figure 1 shows the experimental setup. A commercial clinical ultrasound scanner (Model 3000, Terason, Burlington, MA, USA) was used, with a 7.5-MHz linear-array transducer (Model 10L5, Terason). An MWA device (KY-2000, Nanjing Kangyou Co., Ltd., Nanjing, Jiangsu, China) was used, with a water-cooled MWA needle. Samples of fresh porcine liver were obtained from a local market. Before the experiments, the porcine liver sample was cut to an appropriate size and placed in an acrylic case (6 × 6 × 6 cm^3^) that was filled with a saline solution of 0.9% NaCl. At the bottom of the case, a gel phantom was created to hold the samples and to circumvent the influence that the strong reflection echoes returning from the bottom of the case exerted on the ultrasound RF signals received from the tissue.

### 2.2. Ultrasound Radiofrequency Signal Collection

Firstly, the MWA needle was inserted horizontally into the porcine liver tissue through a circular hole created in a wall of the acrylic case. To prevent the saline solution from leaking from the hole, clay materials were used to plug the hole. The ultrasound transducer was immersed in the 0.9% NaCl saline solution and placed above the porcine liver sample. The distance between the transducer and the sample was determined according to the focal length of the transducer, which was adjustable; thus, the sample could be located in the focal zone of the ultrasound transducer for scanning. The MWA power *P* was set at 80 W and the ablation time *t* at 60 s. Ultrasound RF signals at different time during the ablation procedure were collected consecutively at 2 frame/s using an in-house ultrasound RF signal collection program. The program was developed on the basis of the software development kit (SDK) provided by the Terason system’s manufacturer. After MWA, the porcine liver tissue was cut along the scanning plane of the ultrasound transducer, and gross pathology of final thermal lesions was photographed. The long axis and the short axis of the final thermal lesions were measured and recorded. A total of 15 ex vivo porcine liver samples were ablated in the experiments.

### 2.3. Ultrasound Echo Decorrelation Imaging

The cross-correlation function of two adjacent frames with time interval *τ* is defined as [16]

(1) $$R_{01}\left(r,t\right)=\langle p\left(r,t\right)p^{*}\left(r,t+\tau \right)\rangle$$

where **r** denotes the spatial position vector within the ultrasound imaging plane; *t* denotes time; *p*(**r**,*t*) is the complex analytic signal obtained by conducting Hilbert transform (the Matlab subroutine “hilbert” was used) to the RF signal; the superscript * denotes complex conjugate; and <.> represents the two-dimensional Gaussian convolution of a function *f*(**r**,*t*):

(2) $$\langle f(r,t)\rangle =f(r,t)⊗\exp(-|r{|}^{2}/2\sigma^{2}),$$

where ⊗ is the convolution operator; *σ* is the standard deviation of the Gaussian template. The Matlab subroutine “fspecial (‘gaussian’, hsize, *σ*)” was used for the two-dimensional Gaussian template, where hsize is the size of the Gaussian template. The autocorrelation functions *R*_00_(**r**,*t*) and *R*_11_(**r**,*t*) are defined as

(3) $$R_{00}\left(r,t\right)=\langle {\left|p\left(r,t\right)\right|}^{2}\rangle ;\;\mathrm{and}$$

(4) $$R_{11}\left(r,t\right)=\langle {\left|p\left(r,t+\tau \right)\right|}^{2}\rangle .$$

*R*_00_(**r**,*t*) and *R*_11_(**r**,*t*) can be thought of as maps of integrated backscattered energies at time *t* and *t*+*τ*, respectively. The integrated backscatter *β*(**r**,*t*) is defined as

(5) $$\beta \left(r,t\right)=\sqrt{R_{00}\left(r,t\right)R_{11}\left(r,t\right)},$$

The instantaneous echo decorrelation map is defined as

(6) $$\Delta \left(r,t\right)=2\left[\frac{\beta^{2}\left(r,t\right)-{\left|R_{01}\left(r,t\right)\right|}^{2}}{\beta^{2}\left(r,t\right)+\bar{\beta^{2}\left(r,t\right)}}\right]$$

where $\bar{\beta^{2}\left(r,t\right)}$ is the spatial mean of *β*^2^(**r**,*t*). Logarithmic compression was conducted to Δ(r,t) :

(7) $$\Delta_{\log}\left(r,t\right)=10\log10\left(\Delta \left(r,t\right)\right)$$

The cumulative echo decorrelation image was constructed by taking the maximum of each pixel in instantaneous decorrelation images

(8) $$\Delta_{\log}\left(r\right)=\max\left(\Delta_{\log}\left(r,t_{1}\right),\ldots ,\Delta_{\log}\left(r,t_{i}\right),\ldots ,\Delta_{\log}\left(r,t_{\mathrm{end}}\right)\right),$$

where *t*_1_, …, *t_i_*, …, *t*_end_ correspond to 0.5, 1.0, 1.5, …, 60 s.

### 2.4. Polynomial Approximation

To quantitatively evaluate the final thermal lesions, the polynomial approximation technique [18,19] was applied to the cumulative echo decorrelation image. For completeness, this technique is briefly described as follows. Suppose that the elements in the original cumulative echo decorrelation image before smoothing are *I_x,y_*, where *x* and *y* are indices of the image depth and width, respectively. The function in each direction is assumed to be a polynomial of order *p*. Because *p* is significantly smaller than the number of pixels in the axial and lateral directions, the *I_x,y_* data can be used to determine the optimal polynomial by the least-squares method. *I_x,y_* is then replaced by the value computed by the optimal polynomial. This approximation is performed along each line in the axial and lateral directions. Specifically, let *f_p_*( ·; *V*) be the optimal polynomial of order *p* reconstructed from vector *V* = (*V*_1_, *V*_2_,…, *V_n_*) located at indices 1, 2, …, *n*. Image *J_x,y_* after applying polynomial approximations on the lateral and axial directions is constructed from the following two procedures:

(9) $$I_{x,y}^{*}=f_{p}(x;\;I_{1:n_{d},y}),\;\mathrm{for}\;\mathrm{each}\;y,$$

(10) $$J_{x,y}=f_{p}(y;I_{x,1:n_{w}}^{*}),\;\mathrm{for}\;\mathrm{each}\;x,$$

where *n_d_* and *n_w_* are the numbers of pixels in the axial (depth) and lateral (width) directions, respectively; $I_{1:n_{d},y}$ is the vector *I*_1,*y*_, *I*_2,*y*_,…, $I_{n_{d},y}$; $I_{x,y}^{*}$ is the intermediate image; and *J_x,y_* is the polynomial approximation image.

### 2.5. Ultrasound Radiofrequency Signal Processing

The ultrasound RF signals collected in the ex vivo porcine liver MWA experiments were analyzed. Firstly, the complex analytic signals of two adjacent frames of RF signals with time interval *τ* were obtained using Hilbert transform. In this study, *τ* was equal to 0.5 s. Then, autocorrelation and cross-correlation were calculated using Equations (1), (3), and (4). Finally, the instantaneous and cumulative echo decorrelation images were computed using Equations (6)–(8), and the polynomial approximation images were computed using Equations (9) and (10). The selection of the size of the Gaussian template plays a crucial role in ultrasound echo decorrelation imaging. Fosnight et al. [10] demonstrated that when the size of the Gaussian template is 1/6-1/2 of the size of the final thermal lesion, the decorrelation imaging gets the best performance. In this study, the average size of the final thermal lesions was measured in the experiments, and the decorrelation image noise was the smallest when the size of the Gaussian template was 1/6 of the average size of the final thermal lesions. Therefore, the size of the Gaussian template hsize was chosen as 2.5 × 2.0 mm^2^ (axially × laterally). The standard deviation of the Gaussian template *σ* was set at 1.0 mm. B-mode ultrasound images were also constructed using the envelope of RF signals, obtained by calculating the absolute value of Hilbert-transformed analytic signals. The dynamic range of B-mode images was set at 40 dB. The order *p* for polynomial approximation was set at 5. All the signal processing algorithms were determined using the Matlab software.

## 3. Results

### 3.1. Cumulative Decorrelation Coefficient of Normal Tissue and Thermal Lesions

Regions of interest were selected to calculate the maximum cumulative decorrelation coefficients of normal liver tissues and thermal lesions, respectively. The results showed that the maximum cumulative decorrelation coefficients of normal liver tissues ranged from −0.4 to 0.4, and those of thermal lesions ranged from −1 to −0.4. When a threshold was set at *thr* = −0.4, normal liver tissues and thermal lesions could be distinguished. The areas with the maximum cumulative decorrelation coefficient less than *thr* were determined as normal liver tissues, and those with the maximum decorrelation coefficient greater than *thr* were regarded as thermal lesions.

### 3.2. Detection of Thermal Lesions

**Figure 2:**
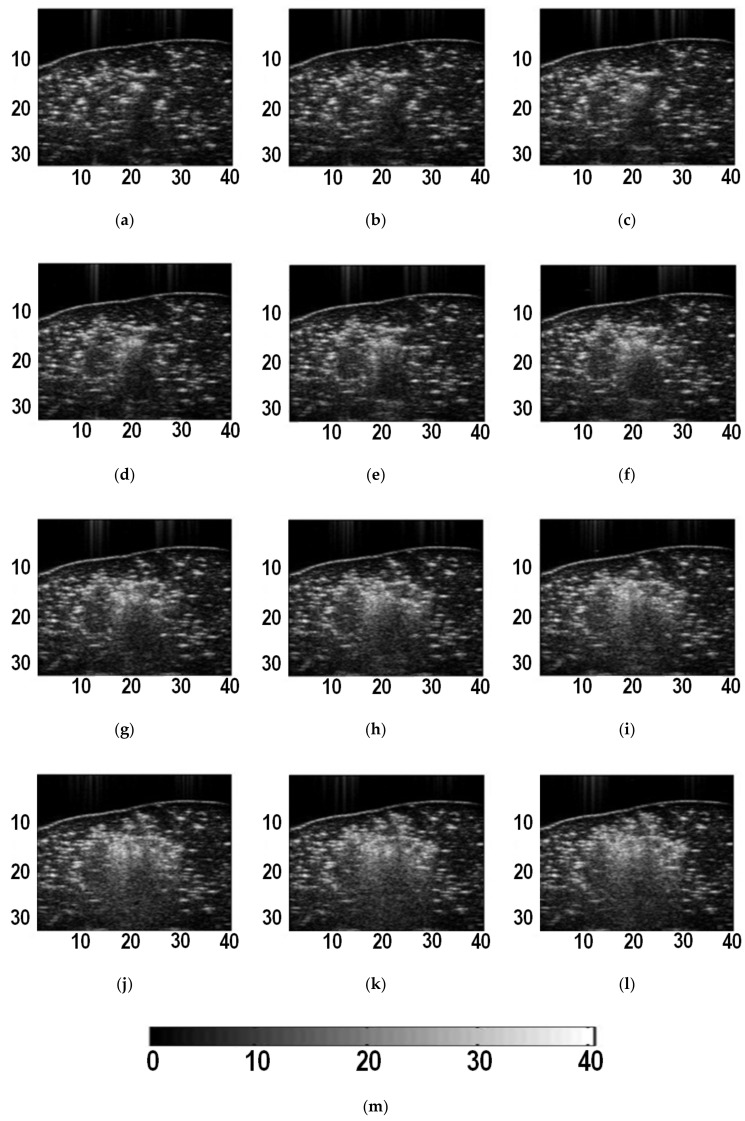
B-mode ultrasound images of liver tissue at different time during ablation. (**a**) 5 s; (**b**) 10 s; (**c**) 15 s; (**d**) 20 s; (**e**) 25 s; (**f**) 30 s; (**g**) 35 s; (**h**) 40 s; (**i**) 45 s; (**j**) 50 s; (**k**) 55 s; (**l**) 60 s. (**m**) is the color bar for (**a**)-(**l**). The units of (**a**)-(**l**) are mm. The unit of (**m**) is dB.

**Figure 3:**
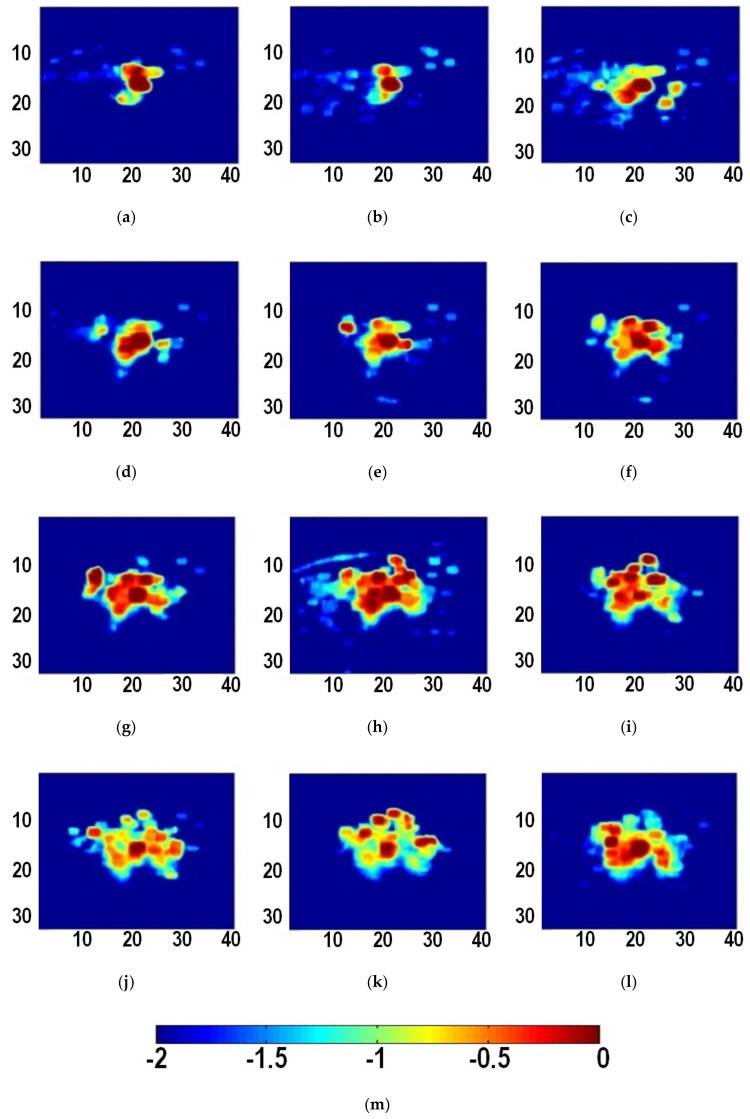
Instantaneous ultrasound echo decorrelation images at different time during ablation. (**a**) 5 s; (**b**) 10 s; (**c**) 15 s; (**d**) 20 s; (**e**) 25 s; (**f**) 30 s; (**g**) 35 s; (**h**) 40 s; (**i**) 45 s; (**j**) 50 s; (**k**) 55 s; (**l**) 60 s. (**m**) is the color bar for (**a**–**l**). The units of (**a**–**l**) are mm. The unit of (**m**) is unitless.

Figure 2 shows typical B-mode ultrasound images of liver tissue at different time during MWA. It can be observed that the heat-induced gas bubbles generated gradually increase the echogenicity within the ablation zone, but such a monitoring method is qualitative and can be affected by the posterior acoustic shadow. The instantaneous echo decorrelation images corresponding to Figure 2 are shown in Figure 3. The instantaneous echo decorrelation images show that the ablation zone increases with increasing ablation time. Compared with B-mode ultrasound imaging, instantaneous echo decorrelation imaging is insusceptible to the influence of gas bubbles and may allow quantitative monitoring of microwave-induced thermal lesions.

**Figure 4:**
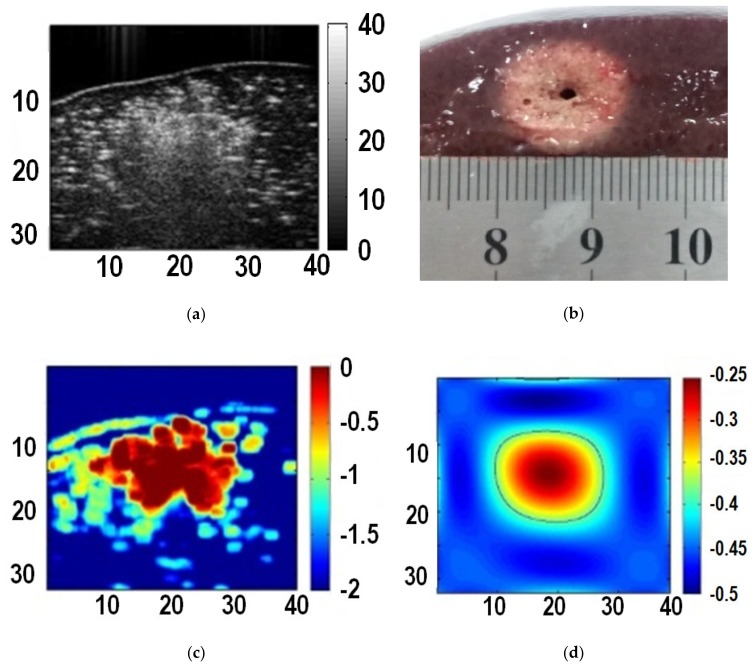
Images of liver tissue at the end of ablation. (**a**) B-mode ultrasound image; (**b**) gross pathology image of the thermal lesion; (**c**) the cumulative echo decorrelation image; (**d**) the polynomial approximation image.

The maximum value of each pixel in the instantaneous echo decorrelation images was taken to form a cumulative echo decorrelation image. According to the analysis in Section 3.1, and the threshold for maximum cumulative decorrelation coefficients *thr* = −0.4 was set experimentally. Subsequently, polynomial approximation [18,19] was conducted to the thresholded cumulative echo decorrelation image to obtain the polynomial approximation image, and the −0.25 iso-contour on the polynomial approximation image was used to quantitatively describe the ablation zone (thermal lesion). Figure 4 shows the B-mode ultrasound image, gross pathology image of the thermal lesion, the cumulative echo decorrelation image, and the polynomial approximation image. It can be seen that the B-mode ultrasound image, affected by acoustic shadow, is not easy to use to describe the thermal lesion. By contrast, the cumulative echo decorrelation image is less affected by the acoustic shadow, and the polynomial approximation image can further quantitatively depict the lesion.

### 3.3. Validation

Gross pathology measurements were taken as the reference standard of thermal lesions. The area of thermal lesions *S* was calculated using the measured long axis *a* and short axis *b*:

(11) S=πab/4.

The −0.25 iso-contour on the polynomial approximation image obtained on the basis of the thresholded cumulative echo decorrelation image was used as the estimated contour of thermal lesions. Then, thermal lesion estimation accuracy was computed by

(12) $$\mathrm{Accuracy}=\frac{\left|S_{2}-S_{1}\right|}{S_{2}}\times 100\%,$$

where *S*_1_ and *S*_2_ denote the areas of the ultrasound-based estimate and the reference standard, respectively. Table 1 shows the validation results of the thermal lesions detected by the proposed method. The average accuracy of the 15 cases was 88.60%, indicating that the proposed method is capable of monitoring the MWA-induced thermal lesions.

## 4. Discussion

In this study, the ultrasound echo decorrelation imaging technique was proposed for monitoring MWA for the first time. The feasibility of monitoring MWA by ultrasound echo decorrelation imaging was proved by the MWA experiments of ex vivo porcine liver.

At present, ultrasound techniques for monitoring thermal ablation are mainly divided into two categories: ultrasound elastography and quantitative ultrasound. Ultrasound elastography is used to monitor thermal ablations because the ablation zone (thermal lesion) is stiffer than normal, untreated tissue [20,21]. However, when heat-induced gas bubbles are generated during the ablation procedure, the bubble-related signals can cause artifacts in ultrasound elastography of ablation zones [22,23,24,25]. For example, the loss of thermal lesion boundary information on ultrasound elastography images was observed in regions in which attenuation occurred because of bubble effects [24]. Thus, ultrasound elastography techniques have been suggested for monitoring procedures that are post-ablation or during ablation before substantial bubbles form [22,23]. Therefore, there are challenges to monitor the intra-operative change of ablation zones using ultrasound elastography.

Quantitative ultrasound (QUS) [26] extracts quantified acoustic parameters from ultrasound backscattered RF signals. Ultrasound echo decorrelation imaging is a kind of QUS parametric imaging techniques. Other QUS methods for monitoring thermal ablations include ultrasound attenuation coefficient [27,28], envelope statistics [19,29,30], mean scatterer spacing (MSS) [31,32,33], and Thermotherapy Ultrasonic View (TUV) [34]. The principle of monitoring ablation zone by ultrasound attenuation coefficient is that the ultrasound attenuation coefficient of ablation zone is higher than that of unheated tissue under the effects of coagulation necrosis and heat-induced gas bubbles [27,28]. The principle of monitoring ablation zone based on envelope statistics distribution model is that the heat-induced gas bubbles increase the scatterer concentration in the ablation zone, and the change of scatterer concentration can be detected by using the shape parameter *m* of the Nakagami model [19,29,30]. MSS is the average spacing between coherent scatterers [31]. Liver lobules are often regarded as coherent scatterers of the liver. The theoretical basis of monitoring ablation zones by MSS is that heating destroys the structure of partial coherent scatterers, resulting in a smaller MSS in ablation zones than in unheated tissues [31,32,33]. TUV is obtained by multidimensional spectral analysis of RF signals, but intra-operative ablation monitoring using TUV can be affected by heat-induced gas bubbles [34].

During the process of thermal ablations, the ablation zone is accompanied by the generation of new bubbles and the disappearance of old bubbles. Both phenomena will lead to the increase of decorrelation of two adjacent frames of ultrasound RF signals. The ultrasound echo decorrelation technique can track the state of bubbles by calculating the decorrelation of two adjacent frames to detect the ablation zone, turning the disadvantageous factor of bubbles into an advantageous factor. Previously, the feasibility of ultrasound echo decorrelation imaging for monitoring RFA and HIFU have been demonstrated. This study further validated the feasibility of ultrasound echo decorrelation imaging for monitoring MWA.

It should be noted that that the polynomial approximation forces a symmetrical behavior around the applicator (microwave ablation needle). Polynomial approximation may be suitable for the ex vivo case where the applicator is operating in an almost homogeneous scenario, but it may lead to imaging artifacts in the clinical application wherein the shape of the region to ablate (and hence the safety region) may have an irregular shape and the tissue may be heterogeneous.

This study has several limitations. First, only ex vivo porcine liver ablation monitoring was carried out using the ultrasound echo decorrelation imaging technique. In vivo animal experiments and clinical studies may be conducted in future work. Second, in the experiments, only the ablation power *P* = 80 W and ablation time *t* = 60 s were used. Note that in cases of larger ablation zones and longer ablation time, the contraction of the tissue undergoing ablation treatments may produce unexpected correlation/decorrelation effects which may affect the imaging results. In future work, more ablation parameters may be tested.

## 5. Conclusions

In this paper, the feasibility of ultrasound echo decorrelation imaging in monitoring MWA was explored using an ex vivo porcine liver model (*n* = 15). Two concluding remarks can be made. (1) Instantaneous ultrasound echo decorrelation imaging is capable to monitor the change of thermal lesions during MWA. (2) Cumulative ultrasound echo decorrelation imaging and polynomial approximation imaging are feasible for quantitatively detecting thermal lesions.

## Figures and Tables

**Table 1:** Validation results of thermal lesions (*P* = 80 W; *t* = 60 s).

No.	Reference Standard (Gross Pathology)			Detection Results				Accuracy (%)
	Long Axis *a*_1_ (mm)	Short Axis *b*_1_ (mm)	Area *S*_1_ (mm^2^)	Long Axis *a*_2_ (mm)		Short Axis *b*_2_ (mm)	Area *S*_2_ (mm^2^)	
1	18.2	13.8	197.16	19.9	14.3		223.39	86.70%
2	15.2	13.3	158.70	16.8	12.6		166.17	95.04%
3	15.4	13.6	164.41	17.7	12.2		169.51	96.90%
4	17.1	12.1	161.47	17.9	13.3		186.88	84.26%
5	17.2	14.2	191.73	19.1	14.1		211.41	89.74%
6	15.7	13.7	168.85	17.4	14.3		190.83	86.98%
7	15.7	13.3	163.92	16.7	14.3		187.47	85.63%
8	14.7	12.7	146.55	13.8	11.7		126.75	86.49%
9	14.9	12.2	142.70	13.5	11.2		118.69	83.71%
10	15.7	13.6	167.61	15.8	12.6		156.28	93.24%
11	16.1	12.4	156.72	15.3	13.7		164.54	95.01%
12	14.7	13.5	155.78	18.3	12.5		179.57	84.73%
13	15.9	12.4	154.77	16.8	12.1		159.57	96.90%
14	14.4	13.2	149.21	19.0	11.9		177.49	81.05%
15	16.3	13.4	171.46	16.7	15.1		197.95	84.55%

## References

[1] Siegel R.L., Miller K.D., Jemal A. (2019). Cancer statistics, 2019. CA Cancer J. Clin..

[2] Ahmed M., Solbiati L., Brace C.L., Breen D.J., Callstrom M.R., Charboneau J.W., Chen M.H., Choi B.I., de Baere T., Dodd G.D. (2014). Image-guided tumor ablation: Standardization of terminology and reporting criteria—A 10-year update. Radiology.

[3] Hoffmann R., Rempp H., Kessler D.E., Weiss J., Pereira P.L., Nikolaou K., Clasen S. (2017). MR-guided microwave ablation in hepatic tumours: Initial results in clinical routine. Eur. Radiol..

[4] Liang P., Yu J., Lu M.D., Dong B.W., Yu X.L., Zhou X.D., Hu B., Xie M.X., Cheng W., He W. (2013). Practice guidelines for ultrasound-guided percutaneous microwave ablation for hepatic malignancy. World J. Gastroenterol..

[5] Asvadi N.H., Anvari A., Uppot R.N., Thabet A., Zhu A.X., Arellano R.S. (2016). CT-guided percutaneous microwave ablation of tumors in the hepatic dome: Assessment of efficacy and safety. J. Vasc. Interv. Radiol..

[6] Scapaticci R., Lopresto V., Pinto R., Cavagnaro M., Crocco L. (2018). Monitoring thermal ablation via microwave tomography: An ex vivo experimental assessment. Diagnostics.

[7] Gertner M.R., Worthington A.E., Wilson B.C., Sherar M.D. (1998). Ultrasound imaging of thermal therapy in in vitro liver. Ultrasound Med. Biol..

[8] Subramanian S. (2015). Thermal Ablation Monitoring Using Ultrasound Echo Decorrelation Imaging. Ph.D. Thesis.

[9] Fosnight T.R., Hooi F.M., Colbert S.B., Keil R.D., Barthe P.G., Mast T.D. (2017). Echo decorrelation imaging of ex vivo HIFU and bulk ultrasound ablation using image-treat arrays. AIP Conf. Proc..

[10] Fosnight T.R., Hooi F.M., Keil R.D., Ross A.P., Subramanian S., Akinyi T.G., Killin J.K., Barthe P.G., Rudich S.M., Ahmad S.A. (2017). Echo decorrelation imaging of rabbit liver and VX2 tumor during in vivo ultrasound ablation. Ultrasound Med. Biol..

[11] Abbass M.A., Killin J.K., Mahalingam N., Mast T.D. (2017). Real-time feedback control of high-intensity focused ultrasound thermal ablation using echo decorrelation imaging. J. Acoust. Soc. Am..

[12] Matsuzawa R., Shishitani T., Yoshizawa S., Umemura S.I. (2012). Monitoring of lesion induced by high-intensity focused ultrasound using correlation method based on block matching. Jpn. J. Appl. Phys..

[13] Sasaki S., Takagi R., Matsuura K., Yoshizawa S., Umemura S.I. (2014). Monitoring of high-intensity focused ultrasound lesion formation using decorrelation between high-speed ultrasonic images by parallel beamforming. Jpn. J. Appl. Phys..

[14] Mast T.D., Pucke D.P., Subramanian S.E., Bowlus W.J., Rudich S.M., Buell J.F. (2008). Ultrasound monitoring of in vitro radio frequency ablation by echo decorrelation imaging. J. Ultrasound Med..

[15] Subramanian S., Schmidt D.T., Rao M.B., Mast T.D. (2016). Dependence of ultrasound echo decorrelation on local tissue temperature during ex vivo radiofrequency ablation. Phys. Med. Biol..

[16] Subramanian S., Rudich S.M., Alqadah A., Karunakaran C.P., Rao M.B., Mast T.D. (2014). In vivo thermal ablation monitoring using ultrasound echo decorrelation imaging. Ultrasound Med. Biol..

[17] Hooi F.M., Nagle A., Subramanian S., Douglas Mast T. (2015). Analysis of tissue changes, measurement system effects, and motion artifacts in echo decorrelation imaging. J. Acoust. Soc. Am..

[18] Wang C.Y., Geng X., Yeh T.S., Liu H.L., Tsui P.H. (2013). Monitoring radiofrequency ablation with ultrasound Nakagami imaging. Med. Phys..

[19] Zhou Z., Wu S., Wang C.Y., Ma H.Y., Lin C.C., Tsui P.H. (2015). Monitoring radiofrequency ablation using real-time ultrasound Nakagami imaging combined with frequency and temporal compounding techniques. PLoS ONE.

[20] Zhou Z., Wu W., Wu S., Xia J., Wang C.Y., Yang C., Lin C.C., Tsui P.H. (2014). A survey of ultrasound elastography approaches to percutaneous ablation monitoring. Proc. Inst. Mech. Eng. Part H J. Eng. Med..

[21] Crocetti L., Calcagni F., Gherarducci G., Tosoratti N., Amabile C., Tarantino F.P., Bargellini I., Cassarino S., Cioni R., Caramella D. (2019). Monitoring of thermal-induced changes in liver stiffness during controlled hyperthermia and microwave ablation in an ex vivo bovine model using point shear wave elastography. Cardiovasc. Intervent. Radiol..

[22] Fahey B.J., Nelson R.C., Hsu S.J., Bradway D.P., Dumont D.M., Trahey G.E. (2008). In vivo guidance and assessment of liver radio-frequency ablation with acoustic radiation force elastography. Ultrasound Med. Biol..

[23] Mariani A., Kwiecinski W., Pernot M., Balvay D., Tanter M., Clement O., Cuenod C.A., Zinzindohoue F. (2014). Real time shear waves elastography monitoring of thermal ablation: In vivo evaluation in pig livers. J. Surg. Res..

[24] Varghese T., Techavipoo U., Zagzebski J.A., Lee F.T. (2004). Impact of gas bubbles generated during interstitial ablation on elastographic depiction of in vitro thermal lesions. J. Ultrasound Med..

[25] Zhou Z., Wu S., Yang C., Tsui P.H. (2015). Stress decay, imaging plane, and gas bubble need to be considered when using ultrasound strain elastography to monitor hepatic ablations. Acad. Radiol..

[26] Mamou J., Oelze M.L. (2013). Quantitative Ultrasound in Soft Tissues.

[27] Zhang S., Xu R., Shang S., Han Y., Liu S., Xu T., Gu C., Zhu X., Niu G., Wan M. (2018). In vivo monitoring of microwave ablation in a porcine model using ultrasonic differential attenuation coefficient intercept imaging. Int. J. Hyperth..

[28] Samimi K., White J.K., Brace C.L., Varghese T. (2017). Monitoring microwave ablation of ex vivo bovine liver using ultrasonic attenuation imaging. Ultrasound Med. Biol..

[29] Zhang S., Shang S., Han Y., Gu C., Wu S., Liu S., Niu G., Bouakaz A., Wan M. (2018). Ex vivo and in vivo monitoring and characterization of thermal lesions by high-intensity focused ultrasound and microwave ablation using ultrasonic Nakagami imaging. IEEE Trans. Med. Imaging.

[30] Zhang S., Han Y., Zhu X., Shang S., Huang G., Zhang L., Niu G., Wang S., He X., Wan M. (2017). Feasibility of using ultrasonic Nakagami imaging for monitoring microwave-induced thermal lesion in ex vivo porcine liver. Ultrasound Med. Biol..

[31] Zhou Z., Wu W., Wu S., Jia K., Tsui P.H. (2017). A review of ultrasound tissue characterization with mean scatterer spacing. Ultrason. Imaging.

[32] Zhou Z., Sheng L., Wu S., Yang C., Zeng Y. (2013). Ultrasonic evaluation of microwave-induced thermal lesions based on wavelet analysis of mean scatterer spacing. Ultrasonics.

[33] Rubert N., Varghese T. (2014). Mean scatterer spacing estimation in normal and thermally coagulated ex vivo bovine liver. Ultrason. Imaging.

[34] Granchi S., Vannacci E., Breschi L., Biagi E. (2018). Advantages of cooled fiber for monitoring laser tissue ablation through temporal and spectral analysis of RF ultrasound signal: A case study. Ultrasonics.

